# Evidence of both foetal inflammation and hypoxia–ischaemia is associated with meconium aspiration syndrome

**DOI:** 10.1038/s41598-021-96275-x

**Published:** 2021-08-18

**Authors:** Kyoko Yokoi, Osuke Iwata, Satoru Kobayashi, Mizuho Kobayashi, Shinji Saitoh, Haruo Goto

**Affiliations:** 1 Department of Pediatrics and Neonatology, Nagoya City University Graduate School of Medical Sciences, 1 Kawasumi, Mizuho-cho, Mizuho-ku, Nagoya, 467-8601 Japan; 2 Department of Pediatrics, Nagoya City University West Medical Centre, Nagoya, Japan; 3 Departments of Diagnostic Pathology, Nagoya City University West Medical Centre, Nagoya, Japan

**Keywords:** Biomarkers, Medical research, Pathogenesis, Risk factors

## Abstract

Foetal hypoxia–ischaemia is a key trigger of meconium aspiration syndrome (MAS). However, many neonates develop MAS without evidence of hypoxia–ischaemia, suggesting the presence of covert but important risk variables. We evaluated the association of MAS with clinical variables, placental histopathologic findings, and inflammatory biomarkers at birth. Of 1336 symptomatic and asymptomatic term singleton neonates with meconium-stained amniotic fluid, 88 neonates (6.6%) developed MAS. Univariate analysis showed that MAS development was associated with low 1- and 5-min Apgar scores, low cord blood pH, funisitis, higher α_1_-acid glycoprotein levels, and higher haptoglobin levels (all p < 0.001 except for p = 0.001 for haptoglobin). Associations of MAS with caesarean delivery (p = 0.004), premature rupture of the membranes (p = 0.006), chorioamnionitis (p = 0.007), and higher C-reactive protein levels (p = 0.008) were lost when adjusted for multiple comparisons. The final multivariate model to explain MAS development comprised lower cord blood pH (odds ratio [OR] 0.58; 95% confidence interval [CI] 0.47–0.73; p < 0.001), funisitis (OR 2.45; 95% Cl 1.41–4.26; p = 0.002), and higher α_1_-acid glycoprotein levels (OR 1.02; 95% Cl 1.01–1.03; p = 0.001). Our data from a large cohort of neonates suggested that intrauterine inflammation is one of the key independent variables of MAS development, together with foetal hypoxia–ischaemia.

## Introduction

Meconium-stained amniotic fluid is common during labour and affects 10–15% of neonates. Approximately 5% of these neonates develop meconium aspiration syndrome (MAS)^1–3^. Despite advances in neonatal resuscitation and respiratory care, MAS remains a serious cause of neonatal mortality and morbidity, especially in developing countries and low-resource settings. It is generally accepted that activation of colonic peristalsis triggered by foetal hypoxia–ischaemia leads to the passage of meconium and foetal gasping movements, resulting in meconium aspiration and respiratory distress^3–6^. Numerous risk factors for MAS and foetal hypoxia–ischaemia have been identified, including post-term pregnancy, foetal distress, low birth weight, mode of delivery, low Apgar scores, and low cord blood pH^7–10^. However, there remain considerable inconsistencies in the relationships among the presence of foetal hypoxia–ischaemia, meconium-stained amniotic fluid, chest radiography findings, and the development of respiratory failure^3^. For example, no evidence of foetal hypoxia and stress is noted in approximately 50% of neonates with MAS^9,11^. This suggests the presence of covert but important risk factors for MAS other than acute foetal hypoxia–ischaemia. Intrauterine inflammation has become increasingly recognised as one of the key variables in the development of MAS^12^. Meconium-stained amniotic fluid is associated with an increased incidence of clinical chorioamnionitis and is accompanied by elevated levels of inflammatory mediators in the amniotic fluid. Interestingly, histopathological chorioamnionitis in term neonates is often observed in the absence of overt bacterial infection^13,14^. The influence of intrauterine inflammation and the subsequent foetal systemic inflammatory response can be evaluated by the histopathological assessment of the umbilical cord and placenta, and previous studies have demonstrated that the presence of chorioamnionitis and funisitis are risk factors for MAS^15^. Consistent with this, we have previously developed a model to explain the development of MAS. The model was based on a cohort of 95 neonates hospitalised in a level-II Neonatal Intensive Care Unit (NICU) with meconium-stained amniotic fluid and comprised birth asphyxia, inflammation, and sex^16^. A study of autopsy cases of MAS highlighted an association between funisitis and villus ischaemic changes, suggesting that the process of lung injury started in utero^17^. Although the association between meconium-stained amniotic fluid and inflammation is well established, the relationship between foetal inflammation and MAS development remains unclear because most previous studies assessed the umbilical cord and placenta in only a part of the study cohort^15,16^.

This study aimed to confirm the relationship between foetal hypoxia–ischaemia, intrauterine inflammation, and the development of MAS in a large cohort of term neonates with meconium-stained amniotic fluid.

## Results

The final study cohort comprised 1336 neonates with meconium-stained amniotic fluid. The neonates were born at 40.2 ± 1.0 weeks of gestation and weighed 3121 ± 411 g at birth. Of these, 88 neonates (6.6%) developed MAS.

Univariate logistic regression analysis showed that the development of MAS was associated with low 1- and 5-min Apgar scores (both p < 0.001), low cord blood pH (p < 0.001) (Table 1), funisitis (p < 0.001), higher α_1_-acid glycoprotein (α_1-_AG) levels (p < 0.001), higher haptoglobin levels (p = 0.001), and higher acute-phase inflammatory reaction (APR) scores (p < 0.001) (Table 2). Associations of MAS development with caesarean delivery (p = 0.004), premature rupture of the membrane (PROM) (p = 0.006), chorioamnionitis (p = 0.007), and higher C-reactive protein (CRP) levels (p = 0.008) were lost when adjusted for multiple comparisons.

**Table 1:** Association of the development of meconium aspiration syndrome and clinical variables among neonates with meconium-stained amniotic fluid on univariate analysis.

	MAS		Odds ratio			p-value
	No	Yes	95% CI			
	N = 1248	N = 88	Mean	Lower	Upper	
Gestation (weeks)	40.20 ± 1.00	40.40 ± 1.04	1.24	0.99	1.55	0.063
Birth weight (g)	3117 ± 400	3154 ± 473	1.00	1.00	1.00	0.413
Birth weight z-score	0.00 ± 1.10	0.05 ± 1.39	1.04	0.86	1.26	0.676
Female sex	571/1248 (45.8)	32/88 (36.4)	0.68	0.43	1.06	0.089
Caesarean delivery	214/1248 (17.1)	26/88 (29.5)	2.03	1.25	3.28	0.004
PROM > 24 h	208/1248 (16.7)	25/88 (28.4)	1.98	1.22	3.23	0.006
Maternal GBS	182/1233 (14.8)	10/86 (11.6)	0.76	0.39	1.50	0.427
Multipara	413/1248 (33.1)	22/88 (25.0)	0.67	0.41	1.11	0.119
1-min Apgar score	8 (0–10)	7 (0–9)	0.57	0.51	0.63	< 0.001
5-min Apgar score	9 (1–10)	8 (2–10)	0.34	0.27	0.42	< 0.001
Cord blood pH	7.25 ± 0.08	7.20 ± 0.12	0.57	0.46	0.70	< 0.001
Cord blood BE	− 6.91 ± 3.20	− 9.82 ± 5.49	0.87	0.83	0.91	< 0.001

Values are presented as mean ± standard deviation, median (quartile ranges) or number (%). Cord blood pH was calculated as per 0.10 pH change.

*CI* confidence interval, *PROM* premature rupture of the membrane, *GBS* Group B streptococcus, *BE* base excess.

**Table 2:** Association of the development of meconium aspiration syndrome, histopathological findings, and inflammatory biomarkers among neonates with meconium-stained amniotic fluid on univariate analysis.

	MAS		Odds ratio			p-value
	No	Yes	95% CI			
	N = 1248	N = 88	Mean	Lower	Upper	
Chorioamnionitis	550/1245 (44.2)	52/88 (59.1)	1.83	1.18	2.83	0.007
Funisitis	661/1245 (53.1)	69/88 (78.4)	3.21	1.91	5.40	< 0.001
**Inflammatory biomarkers at birth**^a^						
C-reactive protein	0.42 (0.29–14.30)	0.70 (0.29–6.90)	1.24	1.06	1.46	0.008
α_1-_AG	26.1 (19–159)	35.2 (19–103)	1.03	1.02	1.04	< 0.001
Haptoglobin	15.2 (12–115)	20 (12–99)	1.02	1.01	1.03	0.001
**Acute-phase inflammatory reaction score at birth**^a^						
0	643/1222 (52.6)	25/88 (28.4)	Reference			< 0.001
1	408/1222 (33.3)	40/88 (45.5)	2.52	1.50	4.22	< 0.001
2 or 3	161/1222 (13.1)	23/88 (26.1)	3.67	2.03	6.64	< 0.001

Values, excluding biomarkers, are expressed as the number (%). Values of biomarkers are presented as the median (range).

Acute-phase inflammatory reaction scores of 0–3 were assigned according to the elevation of C-reactive protein, α_1_-acid glycoprotein, and haptoglobin (see Supplemental Information S4).

*CI* confidence interval, *α*_*1*_*-AG* α_1_-acid glycoprotein.

^a^Cord blood or peripheral venous blood samples collected approximately 1 h after birth.

The final multivariate model to explain the incidence of MAS comprised lower cord blood pH (odds ratio [OR] 0.58 per 0.10 pH change; 95% confidence interval [Cl] 0.47–0.73; p < 0.001), funisitis (OR 2.45; 95% CI 1.41–4.26; p = 0.002), and higher α_1-_AG levels (OR 1.02; 95% CI 1.01–1.03; p = 0.001) (adjusted for gestational age, caesarean delivery, and sex) (Table 3).

**Table 3:** Multivariate logistic model to explain the development of meconium aspiration syndrome.

	Odds ratio			p-value
	Mean	95% CI		
		Lower	Upper	
Gestation (weeks)	1.11	0.88	1.40	0.389
Caesarean delivery	1.47	0.88	2.46	0.142
Female sex	0.68	0.43	1.09	0.107
Cord blood pH	0.58	0.47	0.73	< 0.001
Funisitis	2.45	1.41	4.26	0.002
α_1_-AG	1.02	1.01	1.03	0.001

α_1_-AG was measured from umbilical cord blood or peripheral venous blood samples obtained approximately 1 h after birth.

Cord blood pH was calculated as 0.10 pH change.

*CI* confidence interval, *α*_*1*_-*AG* α_1_-acid glycoprotein.

Alternative multivariate models were developed to assess the potential impact of chorioamnionitis (instead of funisitis) and inflammatory biomarkers other than α_1_-AG on the development of MAS. Model 2, which replaced funisitis with chorioamnionitis, identified cord blood pH and α_1_-AG levels (both p < 0.001), but not chorioamnionitis (p = 0.317), as significant independent variables for MAS development (Supplementary Table S1). Model 3, which replaced α_1_-AG levels with CRP, identified cord blood pH and funisitis (both p < 0.001), but not CRP (p = 0.193), as significant independent variables for MAS development (Supplementary Table S2). Model 4, which replaced α_1_-AG by the APR score (0, 1, and 2 or 3), confirmed cord blood pH (p = 0.001), funisitis (p < 0.001), and APR scores (p = 0.015 and 0.019 for APR scores of 1 and 2 or 3, respectively, compared with score 0) as significant independent variables for MAS development (Supplementary Table S3).

## Discussion

Low cord blood pH, funisitis, and elevated serum α_1_-AG levels at birth were associated with MAS development in a large population of neonates with meconium-stained amniotic fluid. These findings highlight the importance of considering intrauterine inflammation and foetal hypoxia–ischaemia in the mechanism, prediction, and prevention of MAS development.

The pathological mechanism of MAS has primarily been explained by premature bowel peristalsis triggered by intrauterine hypoxia–ischaemia^3–6^. However, studies have identified intrauterine inflammation as an alternative key independent variable for MAS development^12^. Meconium-stained amniotic fluids are associated with intrauterine inflammation, as evidenced by the presence of bacterial contamination and elevated inflammatory biomarkers, such as interleukin-6, in the amniotic fluid^15,18^. The amniotic fluid containing bacteria, microproducts, and proinflammatory mediators, when swallowed by the foetuses, may trigger bowel peristalsis and the passage of meconium in utero, potentially explaining the development of MAS even in the absence of intrauterine hypoxia–ischaemia^15,18,19^. Consistent with these preliminary findings, our study confirmed that the development of MAS is associated with funisitis and serum α_1_-AG levels at birth and foetal hypoxia–ischaemia in a large cohort of neonates with meconium-stained amniotic fluid. However, it should be noted that hypoxia–ischaemia and inflammation often coincide and trigger each other, further rendering the biological response and clinical manifestation complex^20^. Future studies that address the mechanism, early diagnosis, and prevention of MAS may need to consider the role of intrauterine inflammation, as well as hypoxia–ischaemia, in disease pathogenesis.

Univariate analysis showed that funisitis and chorioamnionitis were associated with an increased incidence of MAS in this study. However, when adjusted for other independent variables and covariates, only funisitis, but not chorioamnionitis, was an independent variable for MAS development. Both funisitis and chorioamnionitis are accepted as common consequences of intrauterine inflammation^21,22^. However, there might be subtle but pathologically significant differences between these conditions, such as the stage and extension of intrauterine inflammation and the biological response to the inflammation. Common clinical manifestations of chorioamnionitis include maternal fever, leucocytosis, and foul vaginal discharge, which often proceed to PROM, premature delivery, and intrauterine foetal death. In contrast, clinical symptoms of funisitis appear to represent relatively more foetal conditions, such as leucocytosis, elevated CRP and immunoglobulin M at birth, bronchopulmonary dysplasia, and cerebral palsy^23,24^. Provided that funisitis reflects the foetal response to intrauterine inflammation relatively more specifically (and that chorioamnionitis predominantly reflects the maternal response to inflammation)^25–27^, it would be reasonable to conclude that funisitis is more closely related to the development of MAS than chorioamnionitis. Future studies should address the exact stage and magnitude of intrauterine inflammation related to funisitis and chorioamnionitis.

We used CRP, α1-AG, and haptoglobin as representative serum inflammatory biomarkers at birth in the current study. Univariate analysis results consistently suggested a relationship between the elevation of these biomarkers and the development of MAS. However, only α_1_-AG was identified as an independent variable for MAS development in the multivariate model; no significant improvement of the model was observed when α_1_-AG was replaced by the APR score in Model 4 (Supplementary Table S3). α_1_-AG is a hepatic glycoprotein, the synthesis of which is regulated by glucocorticoids and inflammatory cytokines, such as IL-1, IL-6, and IL-8^28^. An increase in α_1_-AG serum level is associated with various conditions, including cancer, trauma, infection, and inflammation^29^. Simultaneous α_1_-AG and haptoglobin measurement, in addition to CRP, has been proposed for the early detection of systemic infection in neonates^30–32^. CRP levels tend to rise shortly after birth in neonates with MAS, potentially in response to chemical pneumonitis, mechanical ventilation, bacterial superinfection, and airway obstruction^33–35^. In contrast, serum α_1_-AG level slightly increases and remains elevated even after CRP returns to the baseline levels^36,37^. Therefore, α_1_-AG might be suitable for the assessment of chronic inflammation, including intrauterine inflammation. Indeed, in our current study, α_1_-AG levels at birth were more closely associated with MAS development than CRP and haptoglobin. Such a property of α_1_-AG might be relevant as a surrogate marker for intrauterine inflammation associated with histological evidence. Nonetheless, in our study population, there was a trend that an APR score of 2 or 3 was associated with a relatively higher incidence of MAS (Table 2), leaving a possibility that these biomarkers improve the prediction of MAS development in an additive way. Future studies are needed to elucidate whether the combination of CRP, haptoglobin, α_1_-AG, and other inflammatory biomarkers provide a more reliable prediction of MAS development than α_1_-AG alone.

We were able to confirm that both foetal hypoxia–ischaemia and intrauterine inflammation are key independent risk factors for MAS development in a large cohort of neonates. Unlike previous studies, which relied on the clinical diagnosis of chorioamnionitis^25^, our study performed the histopathological assessment of the placenta and umbilical cord and serum biomarker tests in virtually all neonates with meconium-stained amniotic fluid, regardless of subsequent admission to the NICU. This contributed to minimizing selection bias. However, because of the single-centre, retrospective nature of the study, the exact cause-consequence relationships among foetal hypoxia–ischaemia, intrauterine inflammation, and inflammatory response in neonates remain unclear. Because of limited human resources, the placenta and umbilical cord were assessed by a single pathologist using the classifications by Blanc and Redline^38,39^. An alternative standard classification scale, or the Amsterdam Placental Workshop Group Consensus State, provides relatively more detailed information regarding the severity and extent of intrauterine inflammation, which may allow the assessment of relationships between the spatial pattern and severity of funisitis and MAS development^40^. In the multivariate model of our study, together with known independent variables for MAS development, such as gestational age, cord blood pH, and mode of delivery^7–10^, sex was additionally incorporated as one of the covariates based on our preliminary findings, which suggested the dependence of MAS development on sex^16^; however, this relationship was not observed in the current analysis. Considering that our multivariate model does not fully explain the development of MAS, exploration for key independent variables of MAS should be continued.

Our data from a large cohort of neonates suggested that intrauterine inflammation and foetal hypoxia–ischaemia are key independent variables for MAS development. Of the variables supporting the presence of intrauterine inflammation and a foetal biochemical response, funisitis and serum α_1_-AG levels, but not chorioamnionitis or serum CRP and haptoglobin levels, showed robust associations with the development of MAS. These findings may contribute to a more precise understanding of the mechanism underlying MAS development and the prediction, prevention, and treatment of this serious clinical condition. Further studies are needed to elucidate the exact relationship and order between intrauterine inflammation, hypoxia–ischaemia, the elevation of serum biomarkers, and the subsequent development of respiratory failure.

## Materials and methods

This single-centre retrospective observational study was conducted under the approval of the Ethics Committee of Nagoya City University West Medical Centre, Nagoya, Japan (reference number: 18-04-381-451). The need for parental informed consent was waived by the ethics committee (the Ethics Committee of Nagoya City University West Medical Centre) because this study was based on anonymised patients’ data. All methods were carried out in accordance with relevant guidelines and regulations.

### Study population

**Figure 1:**
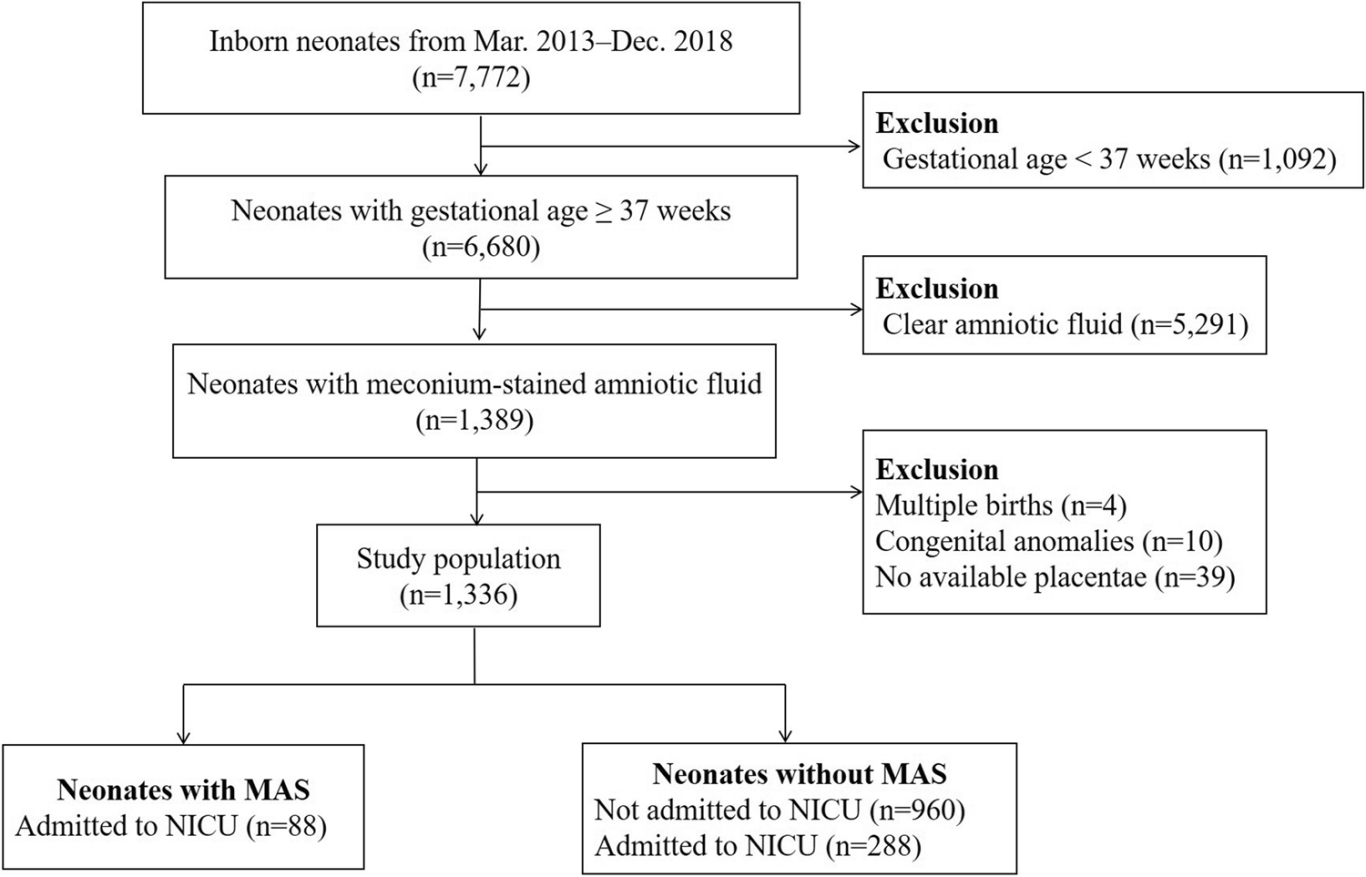
Profile of the study population. *MAS* meconium aspiration syndrome, *NICU* Neonatal Intensive Care Unit.

Between March 2013 and December 2018, a total of 7772 neonates were born at the birth centre of the Nagoya City University West Medical Centre. In the current study, 1389 term neonates with meconium-stained amniotic fluid were reviewed. In this centre, histopathological examination of the placenta is routinely performed for neonates with meconium-stained amniotic fluid. The following neonates were excluded: 39 neonates whose placentae were unavailable and 10 neonates subsequently diagnosed with major congenital anomalies. Additionally, four multiple gestations were excluded because of potential contamination of the amniotic fluid between the siblings. Consequently, 1336 neonates were available for analysis (Fig. 1).

### Clinical variables

The presence of meconium-stained amniotic fluid was visually assessed at the time of the membrane rupture by experienced midwives^10,15,18^. MAS was defined as respiratory distress in neonates born through meconium-stained amniotic fluid, requiring assisted mechanical ventilation or oxygen at a concentration of ≥ 40% for at least 48 h, based on radiographic findings (assessed by an experienced neonatologist, K.Y.)^15,41^. Histopathological examination of the placenta and umbilical cord was performed by an experienced pathologist (M.K.) to diagnose chorioamnionitis and funisitis. Chorioamnionitis was defined as infiltration of neutrophils identified in the chorionic plate using Blanc’s classification (Stage II). Funisitis was defined as infiltration of neutrophils within the walls of the umbilical vessels or in Wharton’s jelly^38,39^.

Patients’ clinical records were reviewed for other clinical variables, including maternal variables (gestational age at delivery, presence of maternal group B streptococcus colonisation, delivery mode, parity, PROM ≥ 24 h, chorioamnionitis, and funisitis). Similarly, neonatal variables, such as birth weight and its z-score, sex, 1- and 5-min Apgar scores, arterial cord blood pH, and other blood tests from the arterial or venous cord or peripheral venous blood at admission, were reviewed.

### Laboratory studies

For neonates with meconium-stained amniotic fluid, umbilical cord blood was obtained to assess serum inflammatory biomarkers, including C-reactive protein (CRP; limit of detection, 0.3 mg/dL), α_1_-acid glycoprotein (α_1-_AG; limit of detection, 20 mg/dL), and haptoglobin (limit of detection, 13 mg/dL), which were routinely checked using a turbidimetric immunoassay (Quick Turbo, Shino-Test Corporation, Tokyo, Japan)^30–32^. In relatively rare cases, when the volume of the cord blood sample was insufficient for the assay, a venous blood sample was additionally obtained approximately an hour after birth. The raw values of these biomarkers and a composite score, or the APR score, of 0 to 3 were calculated according to the elevation of each biomarker above thresholds (see Supplementary Information S4)^32^.

### Statistical analysis

Values are expressed as the mean ± standard deviation, number (%), median (quartile range) or OR; 95% CI. We employed a fully conditional specification using multiple imputations (5 imputed datasets) by the Markov Chain Monte Carlo algorithm to avoid selection bias from missing data^42^. The missing cases for the maternal and neonatal variables, including inflammatory biomarkers, were in the range of 0.1–2.1%. To test the hypothesis that the development of MAS is associated with foetal hypoxia–ischaemia and intrauterine inflammation, a logistic regression model was developed using cord blood pH, inflammatory biomarkers, and histopathological findings as independent variables, adjusting for the priori covariates of sex, delivery mode, and gestational age. Additionally, univariate logistic regression analysis was performed to investigate crude effects of the clinical variables on MAS development, where p-values < 0.003 were considered statistically significant for correcting multiple comparisons for 16 variables. All statistical analyses were performed using SPSS Statistics ver. 26 (IBM Corp, Armonk, NY, USA).

## Supplementary Information

Supplementary Information.
