# Supplementary material for: Evidence of both foetal inflammation and hypoxia–ischaemia is associated with meconium aspiration syndrome
Source: Sci Rep. 2021 Aug 18;11:16799. doi: 10.1038/s41598-021-96275-x (PMC8373916; doi:10.1038/s41598-021-96275-x)
Supplement: Supplementary file 1 — Supplementary Information. [file 41598_2021_96275_MOESM1_ESM.pdf]

Title:

Evidence of both foetal inflammation and hypoxia-ischaemia is associated with meconium aspiration syndrome

Short title: Foetal inflammation and meconium aspiration syndrome

Authors:

Kyoko Yokoi, M.D., Ph.D.[1], Osuke Iwata, M.D., Ph.D.[1], Satoru Kobayashi, M.D., Ph.D.[2], Mizuho Kobayashi, M.D.[3], Shinji Saitoh, M.D., Ph.D.[1], and Haruo Goto, M.D.[2]

Affiliations:

[1]Department of Pediatrics and Neonatology, Nagoya City University Graduate School of Medical Sciences, Nagoya, Japan

[2]Department of Pediatrics, Nagoya City University West Medical Centre, Nagoya, Japan

[3]Departments of Diagnostic Pathology, Nagoya City University West Medical Centre, Nagoya, Japan

Supplementary Table S1: Multivariate logistic model including chorioamnionitis to explain the development of meconium aspiration syndrome (Model 2).

	Odds ratio			P-value
		95% Confidence interval		
	Mean	Lower	Upper	
Gestation (weeks)	1.14	0.90	1.43	0.266
Caesarean delivery	1.50	0.90	2.51	0.123
Female sex	0.68	0.42	1.08	0.098
Cord blood pH	0.59	0.47	0.73	<0.001
Chorioamnionitis	1.28	0.79	2.09	0.317
α_1-acid glycoprotein*	1.02	1.01	1.04	<0.001

Notes: Cord blood pH was calculated as per 0.10 pH change.
*Measured from umbilical cord or peripheral venous blood samples obtained approximately an hour after birth.

Supplementary Table S2: Multivariate logistic model including C-reactive protein to explain the development of meconium aspiration syndrome (Model 3).

	Odds ratio			
		95% Confidence interval		
	Mean	Lower	Upper	P-value
Gestation (weeks)	1.11	0.88	1.40	0.380
Caesarean delivery	1.61	0.97	2.68	0.066
Female sex	0.68	0.43	1.09	0.106
Cord blood pH	0.58	0.47	0.73	<0.001
Funisitis	2.92	1.71	5.01	<0.001
C-reactive protein*	1.12	0.95	1.32	0.193

Notes: Cord blood pH was calculated as per 0.10 pH change.
*Measured from umbilical cord blood or peripheral venous blood samples obtained approximately an hour after birth.

Supplementary Table S3: Multivariate logistic model including the acute-phase inflammatory reaction score to explain the development of meconium aspiration syndrome (Model 4).

	Odds ratio			P-value
		95% Confidence interval		
	Mean	Lower	Upper	
Gestation (weeks)	1.036	0.818	1.313	0.769
Caesarean delivery	1.473	0.879	2.468	0.141
Female sex	0.661	0.415	1.052	0.081
Cord blood pH	2.489	1.421	4.358	0.001
Funisitis	0.583	0.468	0.727	<0.001
Acute-phase inflammatory reaction score at birth*				
0		Reference		
1	1.971	1.139	3.411	0.015
2 or 3	2.171	1.137	4.146	0.019

Notes: Odds ratio for cord blood pH was calculated as per 0.10 pH change.
*Measured using cord blood or peripheral venous blood samples collected approximately one hour after birth. Acute-phase inflammatory scores of 0-3 were assigned according to the elevation of C-reactive protein, α_1-acid glycoprotein, and haptoglobin (see Supplemental Material S1).

Supplementary Information

Supplemental Material S4: Acute-phase inflammatory response score

The acute-phase inflammatory response score is a composite score proposed as an early predictor of severe neonatal infection. Scores 0 (no positive biomarker), 1 (one positive biomarker), 2 (two positive biomarkers), and 3 (all three positive biomarkers) are defined according to the number of positive bed-side inflammatory biomarkers or C-reactive protein (>0.3 mg/dL), α_1-acid glycoprotein (>20 mg/dL), and haptoglobin (>13 mg/dL). Score 3 assessed on day 0-1 is suggestive of severe neonatal infection requiring antibiotic therapies [32].
